# Evaluation of the Effect of Dietary Manganese on the Intestinal Digestive Function, Antioxidant Response, and Muscle Quality in Coho Salmon

**DOI:** 10.1155/2024/9335479

**Published:** 2024-10-28

**Authors:** Dongwu Liu, Wenshuo Xie, Zhiling Xia, Ya Wang, Xinran Zhang, Qiuxiang Pang

**Affiliations:** School of Life Sciences and Medicine, Shandong University of Technology, Zibo 255049, China

**Keywords:** coho salmon, digestion, manganese, muscle quality, superoxide dismutase

## Abstract

Manganese (Mn) is a nutritional element required for fish growth and physiology functions. In this study, we examined the effect of Mn on the intestinal digestive function, antioxidant response, and muscle quality in coho salmon (*Oncorhynchus kisutch*). Nine hundred salmons with initial weight approximately 0.35 g were fed with six isoproteic and isoenergetic diets formulated to contain 2.4, 8.5, 14.8, 19.8, 24.6, and 33.7 mg/kg Mn for 84 days. The result showed that the activity of trypsin and lipase was elevated, whereas *α*-amylase activity was not affected by various Mn diets in intestine. Dietary Mn elevated the activity of Mn-superoxide dismutase (Mn-SOD), total superoxide dismutase (T-SOD), glutathione peroxidase (GSH-PX), and catalase (CAT), but had no influence on copper/zinc-superoxide dismutase (Cu/Zn-SOD) in intestine. Dietary Mn at 8.5, 14.8, 19.8, 24.6, and 33.7 mg/kg enhanced the gene expression level of protein kinase B (Akt) and mammalian target of rapamycin (mTOR). In addition, the accumulation of Mn in muscle was enhanced with increasing levels of dietary Mn. Dietary Mn elevated the content of sodium (Na), potassium (K), magnesium (Mg), and calcium (Ca), but the content of iron (Fe) and Zn was decreased by dietary Mn in the salmon muscle. The content of fatty acids and amino acids was enhanced by various levels of dietary Mn in muscle. Moreover, a significant quadratic effect was observed on the texture of salmon muscle. The dietary Mn requirement was 16.9–25.7 mg/kg Mn to acquire the highest value of muscle texture using the quadratic regression model. The diets at 14.8 and 19.8 mg/kg Mn had a higher score of sensory evaluation for raw muscle. Our result showed that dietary Mn affected the intestinal digestion function and antioxidant response, which may further result in the change of muscle quality in coho salmon. The result will provide reference for detecting the effect of dietary micronutrients on the muscle quality of salmons.

## 1. Introduction

Manganese (Mn) is a nutritional element required for various physiology functions, including reproduction, neuronal function, development, and antioxidant response [1]. As an important nutrient, the role of Mn has been extensively studied for its special function in animals. Particularly, there is a strong relationship between Mn and innate immune and antioxidant response [2–4]. It is known that Mn is a key constituent and activator of metalloenzymes, which could be activated by Mn via a direct combination or an intermediate interaction [5]. Manganese superoxide dismutase (Mn-SOD) is one of the key antioxidant enzymes requiring Mn. Oxidative stress easily generated reactive oxygen species (ROS) in animals, such as hydrogen peroxide (H_2_O_2_) and superoxide anion (O_2_^·−^). However, the overproduction of ROS can be eliminated by antioxidant system, and the primary function of SOD is to detoxify O_2_^·−^ [6]. In animals, the family of SOD includes two kinds of enzymes Mn-SOD and copper/zinc-superoxide dismutase (Cu/Zn-SOD), which bind with different metal cofactors to play functions [7]. To reduce oxidative stress, SOD dismutes O_2_^·−^ to H_2_O_2_, and H_2_O_2_ is further converted into water and oxygen by catalase (CAT) [8]. In the process of breaking down oxidants, Mn is in the reactive catalytic center of Mn-SOD. The antioxidant response may be closely related to Mn homeostasis. Though the function of Mn has been widely investigated [1, 9], the special effort in fish aquaculture needs to be done to reach its beneficial effect.

Environmental stress refers to various adverse factors, such as rapid changes of water temperature, water pollution, insufficient dissolved oxygen, and high aquaculture density [10]. Salmons easily live in the environment of stress, which may result in the stress responses in fish body [11]. It is known that one crucial step of stress is the generation of free radicals, which are a type of highly reactive oxygen-containing molecules produced during metabolic processes. There is a sophisticated antioxidant defense enzyme system to eliminate free radicals and maintain redox balance in fish body. Under environmental stress, the production of ROS significantly increases and the clearance ability is relatively insufficient. The oxidative stress response will be triggered, which will finally cause oxidative damage to cells and tissues of salmons. Thus, it is essential to detect the effect of dietary Mn on coho salmon (*Oncorhynchus kisutch*).

To maintain a stable Mn level for biochemical reactions, the excretion and absorption of Mn need to be tightly controlled in animals [6]. In a healthy diet of fish, Mn is one of the essential nutrients and nutritional supplements. In fish, Mn also plays a crucial role in maintaining the physiology function and development [12]. Dietary Mn is involved in the carbohydrate and lipid metabolism, reproduction, and neurological functions [13]. In addition, the nutrient Mn also regulates antioxidant activities in animals [14]. It has been observed that Mn deficiency inhibits the embryo development and induces skeletal deformity of fish [15–17]. In addition, dietary Mn affects the weight gain, survival, and feed efficiency in fish species [12, 18].

Previously, various studies have been done to investigate the effect of Mn on the aquatic species, which mainly focused on the toxicity, growth, and antioxidant response under Mn exposure [19–22]. However, there were scarce studies on the relationship between SOD activity, digestive function, and muscle quality under the treatment of dietary Mn. It remains unknown whether dietary Mn participates in regulating the digestive function and muscle texture in fish. Trypsin, lipase, and *α*-amylase are main digestive enzymes in the small intestine, which is involved in the process of digesting protein, fat, and sugar in diets [23–25]. Moreover, it is known that the digestive function of intestine and antioxidant states influence the muscle quality. Therefore, it is interesting to investigate the relationship of digestive function, antioxidant states, and muscle texture induced by dietary Mn. The aim of current study was to examine the effect of dietary Mn on the intestinal digestive function, antioxidant response, and muscle quality in coho salmon. The result will provide reference for detecting the effect of dietary micronutrients on the muscle quality of fish.

## 2. Materials and Methods

### 2.1. Diet Formulations

Six isoenergetic and isonitrogenous diets were formulated according to Table 1. Various amounts of Mn sulfate (MnSO_4_ · H_2_O) were added into the basal diets to produce six graded Mn level diets. The final Mn content in diets was 2.4, 8.5, 14.8, 19.8, 24.6, and 33.7 mg/kg Mn after the content of Mn in diets was detected with an inductively coupled plasma emission spectrometer (Agilent 5100, Japan).

### 2.2. Feeding Trial

Coho salmons were obtained from a salmon hatchery factory in Linyi (Shandong, China) and cultured in a flow-through aquarium system. To remove the solid waste and the suspended organic compounds, the system equipped with a microbead filter, a radial flow separator, and a foam fractionator. At the beginning of Mn-dietary experiment, salmons were acclimated for 2 weeks by feeding the control diet in plastic tanks. Then, 900 fish with initial weight approximately 0.35 g were put into 18 240 L plastic tanks. Each experimental diet was randomly assigned to three tanks. The fish was fed to apparent satiation four times daily for 84 days. During the whole experiment, the natural light cycle was adapted. The water temperature and pH was maintained at 16.0 ± 0.5°C and 7.0 ± 0.2, respectively, and ammonia nitrogen was 9.4 ± 0.4 mg/L. The study was carried out according to the recommendations approved by the Experimental Animal Management Methods of Shandong University of Technology.

### 2.3. Sampling Procedures

After 84 days, salmons were fasted for 24 h and 150 mg/L MS222 was used to anesthetize fish. Then, three anterior intestine samples (each sample pooled from three fishes) and four muscle samples (each sample pooled from three fishes) were sampled from each tank for the following analysis. There were 18 anterior intestine samples and 24 muscle samples in total of six dietary Mn treatments.

### 2.4. Analysis ROS Levels, Antioxidant Enzyme, and Digestive Enzyme Activities

The anterior intestine samples were homogenized using a glass homogenizer in the ice cold 0.9% (*w*/*v*) NaCl solution. Then, the activities of Mn-SOD, Cu/Zn-SOD, total superoxide dismutase (T-SOD), glutathione peroxidase (GSH-PX), and CAT, trypsin, *α*-amylase, lipase, and the content of free fatty acids (FFAs) were analyzed with the protocol of Nanjing Jiancheng Bioengineering Institute commercial kits (Nanjing, China). In addition, the content of H_2_O_2_, O_2_^·−^, and protein concentration was analyzed according to the protocol of Beijing Solarbio Science & Technology commercial kits (Beijing, China).

### 2.5. Mn Content and Nutrients Analysis

The muscle samples were added into nitric acid with a previous protocol [26]. Then, the level of Mn and the other elements was detected with an inductively coupled plasma emission spectrometer (Agilent 5100, Japan). The internal standard solution of ^103^Rh obtained from Agilent Technologies was used for the analytical blank solution. Moreover, the quality control was carried out with the blank control and ^103^Rh was used as internal standard.

### 2.6. Fatty Acid and Free Amino Acid Analysis

The content of fatty acids was examined as a previous method [27]. A gas chromatograph (Agilent 7890, USA) was used to detect the content of fatty acids. The samples (approximately 500 mg) were put in 10% sulfosalicylic acid solution and centrifugated at 20,000 rpm for 20 min with a previous method [28]. An automatic amino acid analyzer (Hitachi L-8900, Japan) was used for detecting free amino acids content.

### 2.7. Muscle Texture Measurements and Sensory Evaluation

For texture measurements, the muscle samples were used for detecting muscle quality. A texture analyzer (TA-XT Plus, Stable Micro Systems, Godalming, United Kingdom) was applied for analysis according to a previous protocol [29]. Three samples were used for texture measurements in each dietary Mn treatment. Moreover, a sensory evaluation team consisting of 10 people comprehensively scored and evaluated the color, odor, meat quality, and tissue elasticity of raw muscle samples. The highest score is 10 points and the lowest score is 0 points. A comprehensive score below 5 is considered unacceptable for sensory evaluation.

### 2.8. Real-Time Quantitative Polymerase Chain Reaction (PCR)

Total ribonucleic acid (RNA) of intestine samples was extracted and a PrimeScript RT Reagent Kit (Takara, Japan) was used to transcribe RNA to complementary deoxyribonucleic acid (cDNA). The primer sequences for various target genes as in Table 2. A quantitative thermal cycle (Roche, Lightcycler480, Switzerland) and the SYBR Premix Ex Taq II (Takara, Japan) were used for real-time quantitative PCR. Then, the 2^*−ΔΔCt*^ method was used to analyze the relative gene expression levels.

### 2.9. Statistical Analysis

The statistical analyses were carried out with SPSS 16.0 software (SPSS, Inc., USA). All data were expressed by means ± S.E. One-way analysis of variance (ANOVA) followed by Tukey's test was used to show whether the significant effect (*p*  < 0.05) was present after various dietary Mn treatments. Moreover, the trend analysis was done to detect the quadratic effect on the index of muscle texture, and the optimal dietary Mn requirement was estimated using the quadratic regression model.

## 3. Result

### 3.1. Effect of Dietary Mn on the Digestive Function in the Anterior Intestine

The effect of diets with increasing levels of Mn on the activity of digestive enzymes and FFA content in the anterior intestine was shown in Table 3. The activity of trypsin and lipase was increased (*p* < 0.05) by diets at 14.8, 19.8, 24.6, and 33.7 mg/kg Mn in the anterior intestine (Table 3). In addition, the content of FFA was elevated (*p* < 0.05) by diets at 8.5, 14.8, 19.8, 24.6, and 33.7 mg/kg Mn in the anterior intestine (Table 3). However, the activity of *α*-amylase was not influenced (*p* > 0.05) by Mn in the anterior intestine (Table 3).

### 3.2. Effect of Dietary Mn on the Antioxidant Response in the Anterior Intestine

The effect of diets with increasing levels of Mn on the activity of Cu/Zn-SOD, Mn-SOD, and T-SOD in the anterior intestine of coho salmon was shown in Table 4. Diets at 14.8, 19.8, 24.6, and 33.7 mg/kg Mn elevated (*p* < 0.05) the activity of Mn-SOD in the anterior intestine (Table 4). The highest activity of Mn-SOD was observed at 19.8 mg/kg dietary Mn (Table 4). Nevertheless, no differences were observed (*p* > 0.05) on the Cu/Zn-SOD measured in salmons fed with increasing dietary Mn (Table 4). Diets at 14.8, 19.8, 24.6, and 33.7 mg/kg Mn increased (*p* < 0.05) T-SOD activity in the anterior intestine (Table 4), and the highest activity of T-SOD was observed at 19.8 mg/kg dietary Mn (Table 4). Diets at 8.5, 14.8, 19.8, 24.6, and 33.7 mg/kg Mn enhanced (*p* < 0.05) the activity of CAT and GSH-PX in the anterior intestine (Table 4). However, diets at 8.5, 14.8, 19.8, 24.6, and 33.7 mg/kg Mn enhanced (*p* < 0.05) the level of H_2_O_2_, but decreased (*p* < 0.05) the level of O_2_^·−^ in the anterior intestine (Table 4). The dietary Mn at 19.8 mg/kg induced the highest level of H_2_O_2_ and the lowest level of O_2_^·−^ (Table 4).

### 3.3. Effect of Dietary Mn on the Content of Nutrients in Muscle

The effect of diets with increasing levels of Mn on the content of nutrient elements in the muscle was shown in Table 5. The accumulation of Mn was elevated (*p* < 0.05) with increasing levels of Mn in diets, and the maximum of Mn accumulation was observed at 33.7 mg/kg Mn diet (Table 5). Diets at 8.5, 14.8, 19.8, 24.6, and 33.7 mg/kg Mn increased (*p* < 0.05) the content of sodium (Na; Table 5). In addition, various dietary Mn levels significantly enhanced (*p* < 0.05) the content of potassium (K) and calcium (Ca) in the muscle (Table 5). The content of magnesium (Mg) was increased (*p* < 0.05) by diets at 14.8 and 19.8 mg/kg Mn in the muscle (Table 5). However, the content of iron (Fe) was decreased (*p* < 0.05) by diets at 8.5, 14.8, 19.8, 24.6, and 33.7 mg/kg Mn, and Zn content was decreased (*p* < 0.05) by diets at 14.8, 19.8, 24.6, and 33.7 mg/kg Mn in the muscle (Table 5). In addition, the content of Cu was not influenced (*p* > 0.05) by Mn in the muscle (Table 5).

### 3.4. Effect of Dietary Mn on the Content of Amino Acids in Muscle

The effect of diets with increasing levels of Mn on amino acids in the muscle was shown in Table 6. The content of Thr, Ile, Val, Phe, Lys, Arg, Ser, Ala, and Asp was increased (*p* < 0.05) by diets at 14.8, 19.8, 24.6, and 33.7 mg/kg Mn in the muscle (Table 6). In addition, the content of Met, Tyr, Pro, and Gln was increased (*p* < 0.05) by diets at 8.5, 14.8, 19.8, 24.6, and 33.7 mg/kg Mn in the muscle (Table 6). In addition, the content of Leu was increased (*p* < 0.05) by diets at 8.5, 14.8, 19.8, and 24.6 mg/kg Mn in the muscle (Table 6). Diets at 8.5, 14.8, and 19.8 mg/kg Mn increased (*p* < 0.05) the content of His, and the content of Glu was increased (*p* < 0.05) by diets at 24.6 mg/kg Mn in the muscle (Table 6). However, the content of Gly was not influenced (*p* > 0.05) by Mn in the muscle (Table 6).

### 3.5. Effect of Dietary Mn on the Content of Fatty Acids in Muscle

The effect of diets with increasing levels of Mn on fatty acids in the muscle was shown in Table 7. The content of C14:0, C18:0, C18:1*n*−9, C20:1*n*−9, C20:5*n*−3, and C20:4*n*−6 was increased (*p* < 0.05) by diets at 14.8, 19.8, 24.6, and 33.7 mg/kg Mn in the muscle (Table 7). In addition, the content of C16:0, C16:1*n*−7, C18:3*n*−3, and C22:1*n*−9 was increased (*p* < 0.05) by diets at 8.5, 14.8, 19.8, 24.6, and 33.7 mg/kg Mn in the muscle (Table 7). Moreover, diets at 19.8 and 24.6 mg/kg Mn increased (*p* < 0.05) the content of C22:6*n*−3 in the muscle (Table 7). However, the content of C18:2*n*−6 was not influenced (*p* > 0.05) by Mn in the muscle (Table 7).

### 3.6. Effect of Dietary Mn on the Muscle Texture and Sensory Evaluation

**Figure 1:**
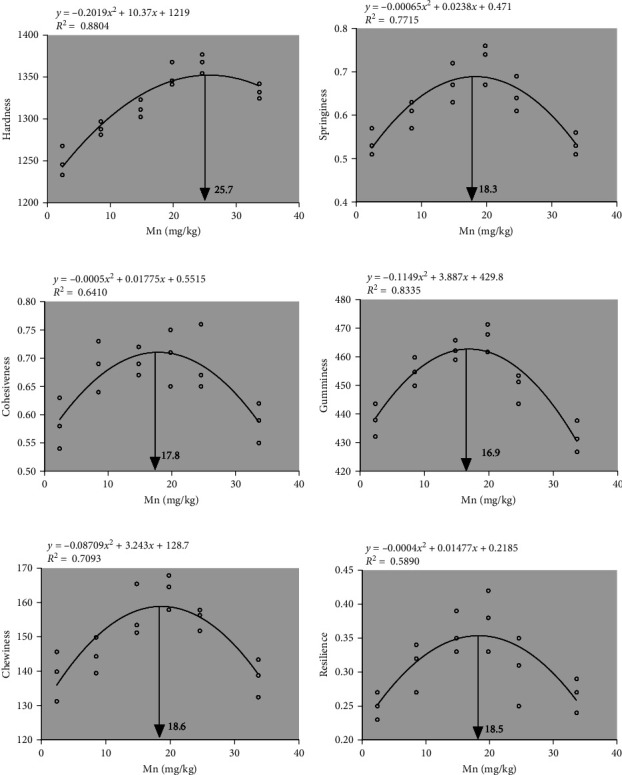
Effect of dietary Mn on the muscle texture of coho salmon. (A) Hardness, (B) springiness, (C) cohesiveness, (D) gumminess, (E) chewiness, and (F) resilience. The trend analysis showed that a significant quadratic effect (*p* < 0.001). The dietary Mn requirement on muscle hardness, springiness, cohesiveness, gumminess, chewiness, and resilience was 25.7, 17.0, 17.8, 16.9, 18.6, and 18.5 mg/kg Mn, respectively, using the quadratic regression model to acquire the highest value of muscle texture. Mn, manganese.

**Figure 2:**
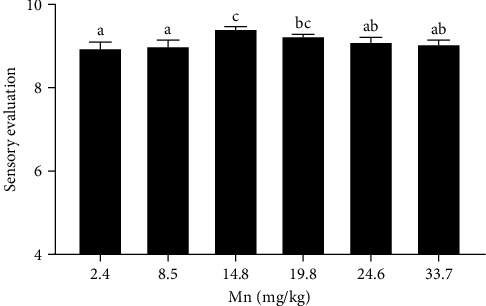
The sensory evaluation of salmon raw muscle. Different letters indicate the significant difference at *p* < 0.05. Mn, manganese.

Effect of dietary Mn on the muscle texture was shown in Figure 1. After various dietary Mn treatments, a significant quadratic effect (*p* < 0.001) was observed on the hardness and springiness (Figure 1A, B). In addition, the cohesiveness, gumminess, and chewiness also showed a significant quadratic effect (*p* < 0.001) after various dietary Mn treatments (Figure 1C,D,E). A significant quadratic effect (*p* < 0.001) was observed on the resilience after various dietary Mn treatments (Figure 1F). The dietary Mn requirement on muscle hardness, springiness, cohesiveness, gumminess, chewiness, and resilience was 25.7, 17.0, 17.8, 16.9, 18.6, and 18.5 mg/kg Mn, respectively, using the quadratic regression model to acquire the highest value of muscle texture. In addition, the score of sensory evaluation of raw muscle at 14.8 and 19.8 mg/kg Mn significantly higher (*p* < 0.05) than that of diet at 2.4 mg/kg Mn (Figure 2). However, no significant changes (*p* > 0.05) was observed after the treatment of 8.5, 24.6, and 33.7 mg/kg dietary Mn (Figure 2). The highest score of sensory evaluation was at 14.8 mg/kg Mn (Figure 2).

### 3.7. Effect of Dietary Mn on Gene Expression Related to Mammalian Target of Rapamycin (mTOR) Signaling in the Anterior Intestine

**Figure 3:**
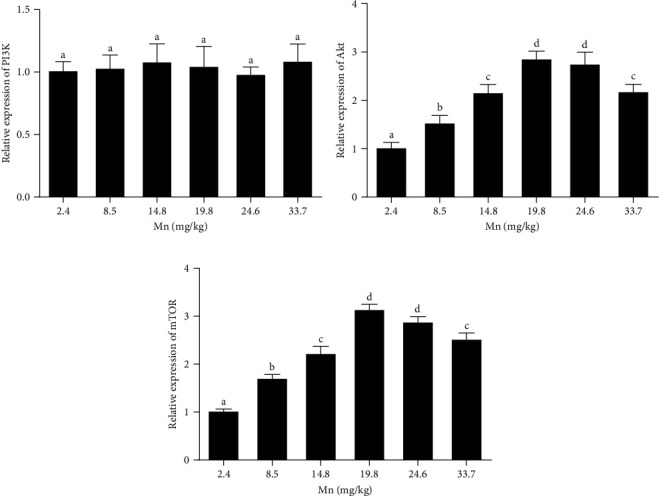
Effect of dietary Mn on the gene expression related to mTOR signaling in the anterior intestine. (A) PI3K, (B) Akt, and (C) mTOR. Different letters indicate the significant difference at *p* < 0.05. Akt, protein kinase B; Mn, manganese; mTOR, mammalian target of rapamycin; PI3K, phosphoinositide 3-kinase.

The effect of diets with increasing levels of Mn on the mTOR signaling in the anterior intestine was shown in Figure 2. No differences were observed (*p* > 0.05) on the phosphoinositide 3-kinase (PI3K) gene expression measured in salmons fed with increasing dietary Mn (Figure 3A). However, diets at 8.5, 14.8, 19.8, 24.6, and 33.7 mg/kg dietary Mn enhanced (*p* < 0.05) the gene expression level of protein kinase B (Akt) and mTOR in the anterior intestine (Figure 3B,C).

## 4. Discussion

The oxidative stress easily induces the overproduction of free radicals and redox reactions in fish tissues [30, 31]. The free radicals may further alter the normal physiology homeostasis and impair the antioxidant defense system to eliminate oxidative stress [32–34]. There are various antioxidant enzymes in this process, including SOD, CAT, and peroxiredoxin (Prdx) [35–37]. As an Mn-SOD component, Mn is involved in the process of antioxidant protection [38]. In the current study, dietary Mn increased the intestinal Mn-SOD activity, and dietary Mn at 19.8 mg/kg resulted in the highest Mn-SOD activity. It suggests that Mn-SOD activity is related to the content of Mn in diet. For Mn-SOD plays a key role in scavenging free radicals, the enhancement of SOD activity can protect intestine against the overproduction of oxygen radicals. It indicates that salmons fed with dietary Mn may be better prepared to deal with oxidative stress. Nevertheless, in this study, dietary Mn did not influence Cu/Zn-SOD in salmons fed with various Mn diets. Previously, divalent Mn treatment induced Mn-SOD activity but it did not influence Cu/Zn-SOD activity in the henrietta lacks (HeLa) cells [39], which is in agreement with the data obtained in this study. In our previous study, we observed that the activity of hepatic Mn-SOD was elevated by dietary Mn in the coho salmons [40]. Thus, dietary Mn helped to avoid the oxidative stress by the induction of Mn-SOD but not Cu/Zn-SOD in salmons.

The ability of removing free radicals is connected with the activities of antioxidant enzymes [41]. It has been found that dietary Mn elevates the antioxidative capacity of fish by the induction of antioxidant enzymes [42]. In the Atlantic salmon, dietary Mn significantly induced SOD activity [43], and SOD can scavenge O_2_^.−^ to H_2_O_2_ [44, 45]. Here, the level of H_2_O_2_ was enhanced but O_2_^.−^ was inhibited with the increase of Mn levels. It showed that the increase of Mn-SOD activity enhanced the removing ability of free oxygen in anterior intestine. The decreased level of O_2_^.−^ is a manifestation of the enhancement of SOD activity.

Trypsin, lipase, and *α*-amylase are main digestive enzymes in the small intestine, which is involved in digesting protein, fat, and sugar in diets [23–25]. Here, the activity of lipase and the content of FFA were increased by dietary Mn in the anterior intestine. Lipase is one of the main digestive enzymes for digesting fat in diets [46–48], the enhancement of FFA content showed that lipase activity is closely related to FFA content [49, 50]. In addition, the content of fatty acids was increased by dietary Mn in the muscle. Thus, the content of saturated and unsaturated fatty acids was enhanced by dietary Mn via the increase of the activity of lipase. In addition, the activities of lipoprotein lipase (LPL), hepatic lipase (HL), and fatty acid synthase (FAS) were elevated by dietary Mn in coho salmons [40]. Thus, the enhancement of lipase activity in intestine may further lead to the lipid deposition in the liver of salmons.

In addition, the activity of trypsin was increased by diets at 14.8, 19.8, 24.6, and 33.7 mg/kg Mn in the anterior intestine. The content of Thr, Ile, Val, Phe, Lys, Arg, Ser, Ala, Asp, Met, Tyr, Pro, Leu, His, Glu, and Gln was increased by dietary Mn in the muscle. The enhancement of nonessential and essential amino acids was the reason of enhancement of trypsin activity in the anterior intestine. However, the activity of *α*-amylase was not affected by various Mn diets in the anterior intestine. It showed that dietary Mn affected the activity of lipase and trypsin but it did not influence the activity of *α*-amylase.

Previously, it is found that Mn accumulation in the fish bone was enhanced with increasing levels of dietary Mn in the grouper and catfish [51, 52]. In the juvenile carp and gibel carp, the accumulation of Mn was also elevated with increasing Mn content [53, 54]. Here, we also found that Mn accumulation in muscle was elevated with the increase of Mn levels, which was consistent with the previous results. Moreover, we observed that the content of Na, K, Mg, and Ca was increased by dietary Mn. However, the content of Fe and Zn was decreased by dietary Mn in the muscle. It showed that the accumulation of Mn affected the nutrient equilibrium in salmon. The level of nutrient elements was related to the content of Mn in diets, and the higher content of Mn had notable effect on the content of nutrient elements in the muscle of salmon.

The muscle texture is a key index to show the muscle quality, and the muscle texture is influenced by the nutritional compositions. The hardness, springiness, cohesiveness, gumminess, chewiness, and resilience are crucial factors influencing fish quality. In our study, after various dietary Mn treatments, a significant quadratic effect was observed on the hardness, springiness, cohesiveness, gumminess, chewiness, and resilience of muscle. The optimal dietary Mn requirement on muscle texture was 16.9–25.7 mg/kg Mn using the quadratic regression model. The enhancement absorption of amino acids and nutrient elements may result in the change of muscle texture, and the muscle texture is closely related to the dietary levels of Mn.

Previously, it is found that the activity of antioxidant enzymes is involved in affecting digestive functions [55, 56]. Here, the activity of trypsin and lipase was elevated but *α*-Amylase activity was not affected by various Mn diets. In addition, the content of fatty acids and amino acids was elevated by various levels of dietary Mn. Moreover, we found that dietary Mn induced the activity of Mn-SOD, CAT, and GSH-PX. Thus, dietary Mn may affect digestive function via the enhancement of antioxidant enzymes in the small intestine of salmon.

It is known that the mTOR signaling is involved in regulating digestive functions [57–59]. In our study, the activity of trypsin and lipase and FFA content was elevated but *α*-amylase activity was not affected by various Mn diets. In addition, the content of fatty acids and amino acids was increased by various levels of dietary Mn. In addition, mTOR signaling is involved in regulating antioxidant enzymes [60, 61]. We found that dietary Mn induced Akt and mTOR gene expression level. Thus, dietary Mn may affect digestive function and antioxidant enzyme activities via mTOR signaling in the small intestine of salmon.

## 5. Conclusion

In summary, the activity of trypsin, lipase, and antioxidant enzymes was elevated by various Mn diets in the intestine. Dietary Mn enhanced the gene expression level of Akt and mTOR. In addition, Mn was accumulated in the muscle with increasing Mn levels, and dietary Mn affected the nutrient equilibrium. The content of fatty acids and amino acids in muscle was elevated by various levels of dietary Mn. A significant quadratic effect was observed on the muscle texture after various Mn treatments. The dietary Mn requirement was 16.9–25.7 mg/kg Mn to acquire the highest value of muscle texture using the quadratic regression model. Our result showed that dietary Mn affected the intestinal digestion function and antioxidant response, which may further result in the change of muscle quality in coho salmon. The result will provide reference for detecting the effect of dietary micronutrients on the muscle quality of salmons.

## Figures and Tables

**Table 1:** Formulation and proximate composition of the experimental diets.

Ingredients (g)	Mn levels (mg/kg)					
	2.4	8.5	14.8	19.8	24.6	33.7
Casein^a^	400.0	400.0	400.0	400.0	400.0	400.0
Gelatin^a^	100.0	100.0	100.0	100.0	100.0	100.0
Dextrin^a^	160.0	160.0	160.0	160.0	160.0	160.0
*α*-Cellulose^a^	95.0	95.0	95.0	95.0	95.0	95.0
Fish oil^b^	75.0	75.0	75.0	75.0	75.0	75.0
Soybean oil^b^	75.0	75.0	75.0	75.0	75.0	75.0
Mineral premix, manganese-free^c^	60.0	60.0	60.0	60.0	60.0	60.0
Vitamin premix^d^	10.0	10.0	10.0	10.0	10.0	10.0
L-Arg	10.0	10.0	10.0	10.0	10.0	10.0
Ethoxyquin	6.0	6.0	6.0	6.0	6.0	6.0
DL-Met	5.0	5.0	5.0	5.0	5.0	5.0
Choline chloride	3.0	3.0	3.0	3.0	3.0	3.0
Ascorbic acid phosphate	0.5	0.5	0.5	0.5	0.5	0.5
Glycine betaine	0.5	0.5	0.5	0.5	0.5	0.5
Manganese sulfate (mg/kg)	0.0	43.0	55.3	61.5	73.7	86.0
Proximate composition						
Crude protein (%)	42.1	42.08	42.04	42.06	42.03	42.05
Crude lipid (%)	12.57	12.53	12.55	12.57	12.59	12.51
Ash (%)	6.12	6.18	6.13	6.17	6.15	6.19
Moisture (%)	7.3	7.31	7.29	7.32	7.28	7.33
Mn (mg/kg)	2.4	8.5	14.8	19.8	24.6	33.7

Abbreviation: Mn, manganese.

^a^Ingredients were obtained: Casein from Sigma Chemical, St. Louis, MO, USA (crude protein 92.24%, crude lipid 0.84%); gelatin from Shandong Yixin Biological Technology Co., Ltd., Shandong Province, China; Dextrin from Shandong Xiwang Sugar Co., Ltd., Shandong Province, China; *α*-Cellulose from Shanghai Lanping Industrial Co., Ltd., Shanghai, China.

^b^Provided by Shandong Conqueren Marine Technology Co., Ltd., Weifang, China.

^c^Composition (mg/kg mineral premix): AlK(SO_4_)_2_ · 12H_2_O, 124.00; CaCl_2_, 17,880.00; CoCl_2_ · 6H_2_O, 49.00; FeSO_4_.7H_2_O, 707.00; CuSO_4_ · 5H_2_O, 32.00; KCl, 1192.00; KI, 5.00; MgSO_4_.7H_2_O, 4317.00; NaCl, 4934.00; Na_2_SeO_3_.H_2_O, 3.00; ZnSO_4_.7H_2_O, 177.00; Ca (H_2_PO_4_)_2_·H_2_O, 12457.00; KH_2_PO_4_, 9930.00.

^d^Composition (IU or mg/kg vitamin premix): retinal palmitate, 10,000 IU; cholecalciferol, 4000 IU; *α*-tocopherol, 75.00 IU; menadione, 22.00 mg; thiamin-HCl, 40.00 mg; riboflavin, 30.00 mg; D-calcium pantothenate, 150.00 mg; pyridoxine-HCl, 20.00 mg; meso-inositol, 500.00 mg; D-biotin, 1.00 mg; folic acid, 15.00 mg; ascorbic acid, 200.00 mg; niacin, 300.00 mg; cyanocobalamin, 0.30 mg.

**Table 2:** Real-time quantitative PCR primers for genes of coho salmon.

Target gene	Forward (5′−3′)	Reverse (5′−3′)	GenBank
PI3K	CCAGTGGCTCAAGGACAAGAACAG	GGATGAAGGTGGCTACGCAGTATC	XM_020466892.1
Akt	GAGTTCACGGCACAGACCATCAC	CGTATGCTGGCGGAGTAAGAGAAC	XM_020503531.1
mTOR	GCAACAGCGACAGCGAGGTAG	TGGAGAGGGAGATTGAGCGGAAG	XM_020506200.1
EF1*α*	ACCGGCCATCTGATCTACAAATGC	CTCTCGCTCAGCCTTCAGCTT	XM_031793751.1

Abbreviations: Akt, protein kinase B; mTOR, mammalian target of rapamycin; PCR, polymerase chain reaction; PI3K, phosphoinositide 3-kinase.

**Table 3:** Effect of dietary Mn on the digestive functions in the anterior intestine.

Enzyme activity and FFA content	Mn levels (mg/kg)					
	2.4	8.5	14.8	19.8	24.6	33.7
Trypsin (U/mg prot.)	2.34 ± 0.22^a^	2.47 ± 0.05^a^	2.91 ± 0.10^b^	3.26 ± 0.17^b,c^	3.53 ± 0.12^c^	3.28 ± 0.19^b,c^
*α*-Amylase (U/mg prot.)	0.20 ± 0.03^a^	0.21 ± 0.03^a^	0.22 ± 0.04^a^	0.23 ± 0.03^a^	0.22 ± 0.03^a^	0.24 ± 0.03^a^
Lipase (U/mg prot.)	4.54 ± 0.13^a^	4.84 ± 0.07^a,b^	4.93 ± 0.07^b^	5.15 ± 0.20^b^	5.02 ± 0.09^b^	5.08 ± 0.20^b^
FFA content (μmol/mg prot.)	0.44 ± 0.03^a^	0.53 ± 0.02^b^	0.55 ± 0.03^b^	0.59 ± 0.03^b^	0.54 ± 0.03^b^	0.53 ± 0.04^b^

*Note*: Values are expressed as means ± s.e.m. (*n* = 3). The different superscript letters (a, b, c) indicate that difference was considered significant at *p* < 0.05.

Abbreviations: FFA, free fatty acid; Mn, manganese.

**Table 4:** Effect of dietary Mn on the antioxidant response in the anterior intestine.

Enzyme activity and FFA content	Mn levels (mg/kg)					
	2.4	8.5	14.8	19.8	24.6	33.7
Mn-SOD (U/mg prot.)	130.49 ± 6.70^a^	146.64 ± 3.24^a,b^	163.02 ± 7.46^b,c,d^	177.15 ± 5.25^d^	164.77 ± 5.48^c,d^	159.87 ± 8.07^b,c^
Cu/Zn-SOD (U/mg prot.)	93.80 ± 3.52^a^	99.45 ± 7.37^a^	95.69 ± 5.08^a^	98.17 ± 5.20^a^	99.50 ± 9.63^a^	95.82 ± 4.10^a^
T-SOD (U/mg prot.)	237.54 ± 7.41^a^	248.95 ± 5.59^a,b^	258.29 ± 7.28^b^	278.16 ± 5.20^c^	260.44 ± 5.46^b^	256.81 ± 4.51^b^
CAT (U/mg prot.)	55.60 ± 2.20^a^	62.79 ± 2.71^b^	67.61 ± 2.27^b,c^	73.11 ± 2.73^c^	68.37 ± 1.51^b,c^	65.50 ± 1.95^b^
GSH-PX (U/mg prot.)	37.59 ± 2.78^a^	47.28 ± 1.35^b^	52.54 ± 2.36^b^	60.94 ± 3.45^c^	52.52 ± 2.42^b^	45.95 ± 2.28^b^
O_2_^·−^ content (U/mg prot.)	55.28 ± 3.55^a^	46.78 ± 1.11^b^	42.08 ± 3.25^b,c^	39.62 ± 1.69^c^	41.40 ± 2.87^b,c^	42.77 ± 1.76^b,c^
H_2_O_2_ content (mmol/mg prot.)	35.05 ± 3.11^a^	42.54 ± 2.44^b^	44.28 ± 1.71^b^	46.05 ± 1.61^b^	41.78 ± 2.34^b^	39.92 ± 2.35^b^

*Note*: Values are expressed as means ± s.e.m. (*n* = 3). The different superscript letters (a, b, c, d) indicate that difference was considered significant at *p* < 0.05.

Abbreviations: CAT, catalase; Cu/Zn-SOD, copper/zinc-superoxide dismutase; FFA, free fatty acid; GSH-PX, glutathione peroxidase; H_2_O_2_, hydrogen peroxide; Mn, manganese; Mn-SOD, manganese-superoxide dismutase; O_2_^·−^, superoxide anion; T-SOD, total superoxide dismutase.

**Table 5:** Effect of dietary Mn on the content of nutrient elements in muscle.

Nutrients content (mg/kg)	Mn levels (mg/kg)					
	2.4	8.5	14.8	19.8	24.6	33.7
Mn	1.17 ± 0.06^a^	1.54 ± 0.08^b^	2.24 ± 0.11^c^	2.73 ± 0.07^d^	3.14 ± 0.11^e^	4.23 ± 0.12^f^
Na	352.86 ± 7.85^a^	376.06 ± 5.03^b^	395.66 ± 4.21^c^	417.76 ± 5.47^d^	427.64 ± 7.25^d^	434.94 ± 8.22^d^
K	1147.35 ± 21.21^a^	1251.72 ± 18.56^b^	1285.76 ± 7.53^b,c^	1315.79 ± 14.96^c^	1317.67 ± 11.41^c^	1147.42 ± 21.58^a^
Ca	212.86 ± 8.57^a^	241.98 ± 6.65^b^	254.40 ± 9.58^b,c^	273.64 ± 5.40^c^	255.47 ± 8.60^b,c^	243.60 ± 9.06^b^
Mg	370.02 ± 5.68^a^	379.74 ± 4.62^a^	412.37 ± 10.66^b^	438.67 ± 6.27^c^	388.03 ± 11.14^a^	366.70 ± 6.21^a^
Cu	3.42 ± 0.28^a^	3.34 ± 0.23^a^	3.23 ± 0.22^a^	3.38 ± 0.28^a^	3.31 ± 0.18^a^	3.53 ± 0.31^a^
Fe	55.94 ± 4.35^a^	44.40 ± 3.21^b^	44.91 ± 3.35^b^	40.07 ± 3.25^b,c^	37.96 ± 5.83^b,c^	32.37 ± 2.85^c^
Zn	24.49 ± 3.23^a^	19.12 ± 1.87^a,b^	15.50 ± 1.21^b,c^	14.78 ± 1.80^b,c^	15.89 ± 1.61^b,c^	12.00 ± 1.63^c^

*Note:* Values are expressed as means ± s.e.m. (*n* = 3). The different superscript letters (a, b, c, d, e, f) indicate that difference was considered significant at *p* < 0.05.

Abbreviations: Ca, calcium; Cu, copper; Fe, iron; K, potassium; Mg, magnesium; Mn, manganese; Na, sodium; Zn, zinc.

**Table 6:** Effect of dietary Mn on the content of amino acids in muscle.

Amino acids (μg/g wet weight)	Mn levels (mg/kg)					
	2.4	8.5	14.8	19.8	24.6	33.7
Thr	105.53 ± 3.79^a^	116.82 ± 4.50^a,b^	122.67 ± 5.59^b,c^	133.40 ± 4.09^c,d^	136.38 ± 4.55^d^	131.52 ± 3.99^c,d^
Ile	23.40 ± 2.07^a^	29.66 ± 1.68^a,b^	32.41 ± 2.23^b^	34.11 ± 3.28^b^	34.13 ± 2.17^b^	29.87 ± 2.23^b^
Leu	46.60 ± 3.30^a^	56.72 ± 3.11^bc^	57.66 ± 0.79^b,c^	61.08 ± 3.34^c^	55.58 ± 1.28^b,c^	53.46 ± 2.86^a,b^
Val	28.60 ± 2.81^a^	33.49 ± 1.10^a,b^	37.37 ± 1.72^b^	38.90 ± 1.28^b^	38.92 ± 2.19^b^	35.26 ± 2.85^b^
Met	18.12 ± 1.82^a^	23.38 ± 2.12^b^	25.02 ± 1.57^b^	24.12 ± 0.77^b^	25.97 ± 0.49^b^	23.34 ± 1.11^b^
Phe	22.74 ± 1.65^a^	27.08 ± 2.68^a,b^	30.38 ± 1.79^b^	31.52 ± 1.80^b^	31.23 ± 1.02^b^	29.59 ± 1.79^b^
Lys	46.04 ± 2.72^a^	52.56 ± 2.51^a,b^	58.11 ± 2.18^b^	57.56 ± 3.35^b^	56.74 ± 2.69^b^	58.05 ± 1.57^b^
His	31.15 ± 1.23^a^	36.03 ± 1.68^b^	35.58 ± 2.22^b^	36.39 ± 1.67^b^	35.18 ± 1.22^a,b^	34.51 ± 1.38^a,b^
Arg	86.05 ± 1.71^a^	86.93 ± 2.13^a^	93.01 ± 2.19^b^	95.23 ± 1.35^b,c^	100.35 ± 2.86^c^	98.13 ± 1.55^b,c^
Tyr	22.68 ± 2.41^a^	28.07 ± 1.54^b^	31.52 ± 1.79^b,c^	34.99 ± 1.58^c,d^	37.08 ± 1.57^d^	35.69 ± 1.11^c,d^
Ser	461.51 ± 5.73^a^	481.26 ± 8.66^a,b^	487.87 ± 9.65^b^	495.92 ± 8.45^b^	490.67 ± 9.43^b^	491.15 ± 13.38^b^
Gly	773.51 ± 15.24^a^	790.56 ± 10.34^a^	799.72 ± 9.09^a^	796.18 ± 11.50^a^	793.29 ± 7.90^a^	782.28 ± 15.31^a^
Ala	533.86 ± 12.72^a^	558.62 ± 8.48^a^	586.33 ± 6.37^b^	588.02 ± 9.44^b^	595.73 ± 11.30^b^	588.91 ± 9.41^b^
Pro	433.78 ± 9.98^a^	461.25 ± 4.49^b^	468.65 ± 7.86^b,c^	475.34 ± 6.80^b,c^	483.96 ± 6.92^c^	467.82 ± 7.90^b,c^
Asp	66.40 ± 2.56^a^	72.66 ± 3.72^a,b^	75.33 ± 1.74^b^	77.55 ± 1.73^b^	76.93 ± 3.78^b^	74.56 ± 2.94^b^
Glu	334.92 ± 11.12^a^	345.67 ± 11.11^a,b^	355.38 ± 10.07^a,b^	352.38 ± 11.06^a,b^	364.97 ± 3.40^b^	351.23 ± 5.56^a,b^
Asn	43.45 ± 2.22^a^	46.76 ± 1.08^a,b^	47.37 ± 2.21^a,b^	51.53 ± 1.80^b,c^	53.52 ± 2.22^c^	43.78 ± 2.80^a^
Gln	147.97 ± 5.90^a^	171.99 ± 4.54^b^	175.31 ± 3.81^b,c^	178.72 ± 4.13^b,c^	188.56 ± 3.42^c^	169.39 ± 6.83^b^

*Note:* Values are expressed as means ± s.e.m. (*n* = 3). The different superscript letters (a, b, c, d) indicate that difference was considered significant at *p* < 0.05.

Abbreviation: Mn, manganese.

**Table 7:** Effect of dietary Mn on the content of fatty acids in muscle.

Fatty acids (mg/kg)	Mn levels (mg/kg)					
	2.4	8.5	14.8	19.8	24.6	33.7
C14:0	1.22 ± 0.11^a^	1.36 ± 0.09^a,b^	1.48 ± 0.08^b,c^	1.64 ± 0.06^c^	1.54 ± 0.06^b,c^	1.49 ± 0.08^b,c^
C16:0	4.32 ± 0.10^a^	4.54 ± 0.06^b^	4.67 ± 0.07^b,c^	4.79 ± 0.09^b^	4.57 ± 0.06^c^	4.52 ± 0.04^b^
C18:0	2.26 ± 0.12^a^	2.43 ± 0.12^a,b^	2.53 ± 0.11^b^	2.63 ± 0.03^b^	2.60 ± 0.06^b^	2.56 ± 0.03^b^
C16:1*n*−7	1.93 ± 0.18^a^	2.23 ± 0.11^b^	2.35 ± 0.06^b^	2.45 ± 0.04^b^	2.44 ± 0.04^b^	2.41 ± 0.07^b^
C18:1*n*−9	3.17 ± 0.07^a^	3.35 ± 0.10^a,b^	3.45 ± 0.06^b,c^	3.58 ± 0.04^c,d^	3.66 ± 0.05^d^	3.52 ± 0.09^b,c,d^
C20:1*n*−9	0.88 ± 0.09^a^	0.96 ± 0.06^a,b^	1.07 ± 0.04^b^	1.11 ± 0.04^b,c^	1.32 ± 0.08^d^	1.27 ± 0.04^c,d^
C22:1*n*−9	0.49 ± 0.05^a^	0.63 ± 0.04^b^	0.66 ± 0.04^b^	0.71 ± 0.04^b^	0.70 ± 0.03^b^	0.64 ± 0.03^b^
C18:2*n*−6	7.44 ± 0.13^a^	7.75 ± 0.08^a^	7.83 ± 0.07^a^	7.54 ± 0.21^a^	7.83 ± 0.15^a^	7.64 ± 0.26^a^
C18:3*n*−3	1.24 ± 0.08^a^	1.41 ± 0.04^b^	1.52 ± 0.06^b,c^	1.57 ± 0.06^c^	1.64 ± 0.03^c^	1.63 ± 0.04^c^
C20:5*n*−3	0.28 ± 0.06^a^	0.36 ± 0.03^a,b^	0.42 ± 0.03^b^	0.62 ± 0.05^c^	0.65 ± 0.04^c^	0.55 ± 0.04^c^
C20:4*n*−6	0.35 ± 0.04^a^	0.39 ± 0.03^a,b^	0.45 ± 0.04^b,c^	0.51 ± 0.03^c,d^	0.59 ± 0.04^d^	0.54 ± 0.03^c,d^
C22:6*n*−3	4.66 ± 0.10^a^	4.73 ± 0.05^a,b^	4.79 ± 0.04^a,b^	4.84 ± 0.03^b,c^	4.93 ± 0.03^c^	4.80 ± 0.05^a,b,c^

*Note*: Values are expressed as means ± s.e.m. (*n* = 3). The different superscript letters (a, b, c, d) indicate that difference was considered significant at *p* < 0.05.

Abbreviation: Mn, manganese.

## Data Availability

The data are available from the corresponding author upon reasonable request.
